# Survival Strategies of Duckweeds, the World’s Smallest Angiosperms

**DOI:** 10.3390/plants12112215

**Published:** 2023-06-03

**Authors:** Paul Ziegler, Klaus J. Appenroth, K. Sowjanya Sree

**Affiliations:** 1 Department of Plant Physiology, University of Bayreuth, 95440 Bayreuth, Germany; paul.ziegler@uni-bayreuth.de; 2 Matthias Schleiden Institute—Plant Physiology, University of Jena, 07743 Jena, Germany; klaus.appenroth@uni-jena.de; 3 Department of Environmental Science, Central University of Kerala, Periye 671320, India

**Keywords:** abiotic stress, biotic stress, duckweed, Lemnaceae, turion

## Abstract

Duckweeds (Lemnaceae) are small, simply constructed aquatic higher plants that grow on or just below the surface of quiet waters. They consist primarily of leaf-like assimilatory organs, or fronds, that reproduce mainly by vegetative replication. Despite their diminutive size and inornate habit, duckweeds have been able to colonize and maintain themselves in almost all of the world’s climate zones. They are thereby subject to multiple adverse influences during the growing season, such as high temperatures, extremes of light intensity and pH, nutrient shortage, damage by microorganisms and herbivores, the presence of harmful substances in the water, and competition from other aquatic plants, and they must also be able to withstand winter cold and drought that can be lethal to the fronds. This review discusses the means by which duckweeds come to grips with these adverse influences to ensure their survival. Important duckweed attributes in this regard are a pronounced potential for rapid growth and frond replication, a juvenile developmental status facilitating adventitious organ formation, and clonal diversity. Duckweeds have specific features at their disposal for coping with particular environmental difficulties and can also cooperate with other organisms of their surroundings to improve their survival chances.

## 1. Introduction

Duckweeds are small, simply constructed aquatic higher plants or macrophytes that represent an extreme and highly successful adaptation to life on or just below the surface of quiet fresh water. Their integration into the realm of vascular aquatic plants [1] and their anatomical, morphological, physiological, ecological, and distributional features have long been described [2,3] and recently revisited [4]. Particular anatomical and physiological features enable them to grow and maintain themselves on ponds, ditches, slowly flowing streams, and other small bodies of water worldwide in all climate zones. The success of duckweeds in colonizing and persisting on quiet water surfaces is based on extensive reduction of the well-developed root and shoot systems that are characteristic of most higher plants for taking up nutrients and for exposing assimilation and reproductive organs to light and the airspace. Duckweed individuals consist primarily of leaf-like assimilatory organs, or fronds. The duckweed frond is a thallus-like structure of less than 1 to 15 mm in diameter or length and only a few cells in thickness that represents a fusion of leaves and stem and, thus, the extreme reduction of an entire vascular plant. The fronds consist largely of spongy mesophyll with large air spaces that make them buoyant, and they are either rootless or bear one to several simple hairless adventitious roots on the underside. Duckweeds are thought by some groups of researchers to represent a subfamily (Lemnoideae) of the Araceae (see [5] for a new publication), and this has also been suggested by the Angiosperm Phylogeny III decision. However, our research indicates that duckweeds better constitute a family (Lemnaceae) in its own right and that this is also in agreement with basic taxonomic rules [6]. Although the Lemnaceae have been considered until recently to consist of 37 species (e.g., [7]), the number of species has recently been revised to 36 [8]). These species are distributed among five genera (*Spirodela*: abbreviation *S.*, *Landoltia*: *La.*, *Lemna*: *Le.*, *Wolffiella*: *Wa.*, *Wolffia*: *Wo.*), which differ in the size and complexity of the fronds and in the number of roots they bear [2,4,7,9,10,11,12,13]. The differences reflect an evolutionary progression from *Spirodela* to *Wolffia* in terms of morphological reduction and genome augmentation [4,14].

Despite their small size, simple structure, and inconspicuous appearance, duckweeds are widespread on Earth, inhabiting all climate zones except the very cold polar regions and extremely dry deserts. Some species are quite cosmopolitan, such as *S. polyrhiza* and *Le. aequinoctialis*, which inhabit all continents, whereas others are confined to certain continents (e.g., *Wo. brasiliensis* in both North and South America), to a single continent (e.g., *Le. perpusilla* in North America), or to much more restricted regions (e.g., *Le. tenera* in southeast Asia, *Wa. denticulata* in southeast Africa, and *Wo. microscopica* in the northern part of the Indian subcontinent) [2,7,12]. Multiple duckweed species can inhabit particular regions: six different species have been identified in each of China, the Ukraine and Israel [15,16]. Whatever the regions inhabited by the various species, the ability of duckweeds to successfully colonize compatible water bodies and to persist in these habitats is due in large part to pronounced growth potential, juvenile organization, and clonal vegetative propagation. These attributes, together with small size and floating habit, provide duckweeds with a unique means to productively respond to environmental challenges.

### 1.1. Growth and Vegetative Propagation

The restriction of duckweeds to small floating assimilatory organs facilitates rapid growth. Duckweed fronds consist mainly of photosynthetic tissue, and the channelling of produced photosynthate into the production of new, simply constructed photosynthetic tissue constitutes streamlined utilization resulting in rapid augmentation of frond biomass. Indeed, duckweeds have been shown to be the most rapidly growing higher plants in laboratory experiments [17,18] and produce large amounts of biomass under natural conditions and in agricultural/industrial contexts that can be utilized for, e.g., bio-energy production [3,4,9,10,11,19]. This strong growth potential is coupled with vegetative propagation to result in rapid frond production. Although duckweeds can, in principle, flower, and some indeed do so regularly, the main means of propagation of all duckweed species is the budding of daughter fronds from one or two pouches in the mother fronds while remaining attached for a time via stipes to form colonies of 2 to 50 connected fronds [2,4,10,11,20]. The growth of duckweeds is, therefore, often quoted as an increase in frond number, as well as an increase in frond weight. Rapid growth of duckweeds thus manifests itself in the production of numerous colonies of interconnected fronds that spread out over the water surface. Frond colonies tend to distribute themselves equidistantly over free water surfaces, probably by exuding surface-active repellent substances into the surrounding water, thus ensuring optimal access to water nutrient substances [9]. The potential for rapid growth and the vegetative reproduction of fronds and frond collectives (colonies) enables duckweeds to successfully colonize stretches of quiet open water without having to resort to time- and internal resource-consuming sexual propagation.

### 1.2. Juvenile Organization and Adventitious Development

The vegetative propagation of duckweeds can be understood in the context of restriction of development to a juvenile stage and adventive organ formation. The lack of differentiation of the duckweed assimilatory axis or “shoot”, i.e., the frond, into the distinct classical shoot and leaf systems is reminiscent of embryonal or seedling organization, and the organs developing from this juvenile shoot (quite generally daughter shoots or fronds, but also roots, flowers, and bracts, when present) can be regarded as irregularly formed or adventive organs [9]. Along with the small size of the duckweed frond, the simplified juvenile structure to be reproduced is an important factor in enabling the rapid propagation of duckweed fronds. In addition, the adventitious propagation of juvenile assimilatory shoots can be readily modified upon the impact of appropriate signalling to enable the development of frond derivatives that can help a duckweed cope with highly unfavourable climatic conditions. This is evident in the formation of resting fronds for overwintering and of flowers for the production of seeds, to be discussed in the following.

### 1.3. Clonal Diversity

The vegetative propagation of duckweed fronds gives rise to clones of the mother fronds, i.e., all the progenies of a particular frond have the same genetic makeup as the mother frond. However, clonal diversity is a characteristic of duckweeds, which becomes evident when certain attributes are compared among isolates of a single species collected from different geographical regions. Not only species themselves but also different clones of individual species can show considerable variation in growth potential [17,18], salt tolerance [21], the ability to accumulate starch under nutrient deficiency [22], and the ability to grow on agricultural wastewater [23]. Even the genotypes of 22 of 23 investigated clones of *S. polyrhiza* could be differentiated by several orthogonal genotyping methods [24]. Clonal differences in the specific turion yields of *S. polyrhiza* have been found to be stable after years of in vitro cultivation and are assumed to be genetically determined [25]. However, intraspecific genetic diversity in *S. polyrhiza* is extremely low, in association with a low mutation rate [26,27]. Clonal diversity thus represents a largely asexual adaptation to different surroundings and may be an example of epigenetic acclimation as an alternative to adaptation through natural selection [28]. Stress-induced DNA methylation can be an important factor in the epigenetic background of clonal diversity [29], which may be enhanced by spontaneous polyploidization that can create a fitness increase for some already existent strains in some stressful environments [30].

## 2. Duckweed Survival

As small, free-floating aquatic plants, duckweeds can easily be displaced or removed from their habitat by the action of moving water and wind, foraging by water animals, and gathering by man. They are also susceptible to incapacitation or destruction of their habitat by the impact of unfavourable environmental conditions such as excessive cold, water pollution, or competition for the water surface (see [2]). A quite fundamental factor in ensuring duckweed survival in general is thus the ability to establish themselves in new surroundings. This requires the ability to reach these new surroundings and then proliferate in them.

Although duckweeds have the potential to grow and propagate themselves rapidly, they can only do so under propitious, non-limiting conditions. These include favourable temperatures, adequate lighting conditions, a sufficient supply of mineral salts, and a lack of serious competition for the water surface. When these requirements are met in nature—as they are to at least some extent during the growing seasons of the various climate zones—duckweeds can successfully colonize their surroundings. However, numerous factors can encroach upon these favourable constellations to impede or even stop growth and propagation or to damage or even kill the fronds. Insufficient mineral salt nutrition and low temperatures can severely curtail growth and metabolism; frost and desiccation can be lethal to the fronds. The surface of the water body can be overgrown by other macrophytes and by duckweeds themselves; the fronds can be subject to microbial attack and exposed to toxic water-contaminating substances.

In the context of their adaptation to life on the water surface in many different climate zones, duckweeds have evolved structural and physiological features and developmental patterns that go beyond the mere potential for rapid growth and serve to cope with the manifold influences that can compromise growth and propagation. These attributes are discussed in Section 4 in terms of how duckweeds can maintain their distribution status on the water surface during the growing season in the face of adverse influences. These include coping with the prevailing temperature and light regimes, ensuring sufficient nutrition, resisting microbial attack and cooperating productively with aquatic microorganisms, and coping with overcrowding and water pollution. On the other hand, the means by which duckweeds can withstand conditions that effectively preclude any growth at all and can be lethal to the organisms are discussed in Section 5 and Section 6. The normal growing season fronds of duckweeds faced with critical conditions best exemplified by winter cold can produce quiescent “resting” fronds that can tolerate and “wait out” the unfavourable conditions and resume “normal” vegetative growth and propagation when conditions improve. Duckweeds can withstand drought—along with other unfavourable conditions—by flowering and forming resilient seeds that can later germinate to form a new, sexually recombinant generation of fronds under appropriate conditions.

## 3. Colonization of New Habitats

The colonization of new habitats by duckweeds depends on the ability to disperse from already-occupied habitats, proliferate in newly reached habitats, and compete with already-established species there [31].

Duckweed fronds growing at a particular location on a water body can be transported to another part of the water body or to another water body by water currents, flooding, wave action, and being blown across the water by wind. However, the main means of duckweed relocation is via adherence to animals that live in or near water, such as muskrats, and especially birds [2,32]. This dispersal is facilitated by the small size of duckweed fronds, but especially transport out of the water may be limited by the inability of the fronds to survive severe desiccation [2,31,32,33]. Nevertheless, *Le. minuta* fronds were found to retain moisture and viability for a prolonged period between duck feathers, supporting the idea of epizoochorous transport by birds [34]. Transport by birds can also occur by endozoochory, as fronds of *Wo. columbiana*, *Le. minor,* and *Le. gibba* were found to survive passage through the guts of waterfowl [35,36]. In time, repeated short-range relocation events can result in far-reaching dispersal of the fronds [2,37], and long-distance dispersal by birds may also occur [7,38]. Duckweed seeds can, in principle, be transported in the same ways as fronds, but the tendency of the seeds to sink to the bottom of the water body and the predominantly vegetative propagation of duckweed fronds indicate only a minor role for seed relocation and dispersal in the colonization of new habitats.

When duckweed fronds have arrived at a new location, they must be able to propagate rapidly to successfully establish themselves in the new habitat. This can be achieved based on the pronounced growth potential and clonal vegetative reproduction characteristic of duckweeds under conditions of sufficient mineral salt nutrition and light, favourable temperature, sufficient water space on the surface, lack of toxic water substances, and lack of serious competition. Specific growth potential may determine success in colonizing new water bodies when conditions are otherwise comparable: the higher growth rate of *Le. minor* in comparison with *Le. trisulca* was regarded to be decisive in the far greater frequency of the former in regions where both species were distributed [31]. However, the degree to which superior growth potential can be realized upon interaction with the environment has been only anecdotally investigated. Nutrient availability is a primary factor in enabling a duckweed to establish itself on the surface of a water body, with especially nitrogen driving the initial phases of clonal expansion of *Le. minor* [39,40]. Light availability strongly interacts with nutrient availability in determining *Le. minor* dominance of the water surface [41]. Duckweeds must be able to survive in regions characterized by seasons of particularly harsh conditions. An example is the ability of frost-sensitive *S. polyrhiza* fronds to survive freezing winter temperatures by developing frond derivatives (turions) that can withstand the cold season in contrast to the closely related *S. intermedia* with equally frost-sensitive fronds that do not develop turions [38]. Success in colonization is also dependent on the absence of potentially lethal seasonal developments such as summer increases in water pH to values above 8 [31].

The consolidation of the colonization of a new habitat by a duckweed, i.e., persistence on the newly occupied water body, depends on the ability of the duckweed to ensure the growth and propagation required to maintain the duckweed population during the growing season. This includes coping with high temperatures, low and high light intensities and nutritional deficiency, competing successfully with other aquatic plants, resisting attack by microorganisms, productive cooperation with aquatic microbiota, and withstanding the presence of harmful substances in the water (see Section 4). Duckweeds must also be able to cope with low temperatures and drought that prevent growth and may be life-threatening if they are features of the inhabited region. This includes the production of resting fronds to withstand the cold season (see Section 5) and flowering and the production of seeds to avoid severe drought (Section 6). Examples of successful colonization by duckweeds are provided by alien invasive species such as *Le. minuta*, which has spread across much of western and central Europe in the past 6–7 decades [42], or *Le. aequinoctialis*, which has recently migrated into the Ukraine [43]. Substantial genetic diversity exhibited by *Le. minuta* having colonized Ireland is thought to reflect repeated invasions across an extensive open-water barrier from continental Europe [44]. Studies of *Le. minuta* have illustrated some ways in which this alien species asserts itself against the resident duckweed *Le. minor* upon its arrival at new locations. *Le. minuta* was found to make better use of high light intensities than *L. minor* [45] and to be more tolerant of drought and the presence of metals in the water [46]. The latter study indicated, however, that the relative performances of an alien and a native species depend on multi-faceted differences between the species and on the nature of the stressors that are involved.

## 4. Coping with the Growing Season

If a duckweed has established itself in a given environment, its growth and propagation must be compatible with the prevailing temperatures, light regime, nutrient supply, and pH value of the water. In addition, the duckweed must be able to fend off a microbial attack or at least contain it to an acceptable extent, tolerate the presence of harmful substances in the water, and assert itself in the face of intra- and interspecific competition for light, nutrients, and space. The ability of the various species of duckweed to adapt to widely differing regimes of temperature, light, nutrient availability, and medium pH is basically rooted in the specific attributes of the species in question that have developed in the course of the evolution of that particular species. The extent of the ability of a particular species to tolerate changes in its environmental parameters is also basically delineated by the characteristic attributes of the species. The adaptive and tolerative potential of a duckweed species is further diversified by the clonal diversity that the species can exhibit. The ability of a species to resist a microbial attack can similarly be enhanced by clonal diversity. The ability of duckweeds to adapt to an environment and tolerate its potentially harmful influences can include active metabolic reactions. This can take the form of measures to combat the ill effects of excessive light, the presence of harmful substances in the water, and competition. Duckweeds also actively contribute to the interaction with aquatic microorganisms that can be beneficial to both organisms. The sum of the species-specific adaptive attributes diversified by clonal variation and inducible reactions to cope with harmful influences and to promote mutualism are vital for duckweed survival.

### 4.1. Temperature, Light, and pH

#### 4.1.1. Temperature

Duckweed growth results from many interacting temperature-dependent chemical processes, including nutrient uptake, nutrient assimilation and transport, photosynthesis, and respiration, as well as many other processes incorporating enzymatic activities [3]. The survival of a duckweed under a particular temperature regime depends on the genetically determined intrinsic ability of the organism to grow well and propagate at the temperatures in question that have evolved with the formation of the species and its clonal derivatives. The optimum temperatures for the growth of numerous duckweed species and clones were found to vary between 20 °C and 30 °C; minimum temperatures that just enable a very slow permanent growth rate were found to range between <8 °C and 16–20 °C, and long-term maximum temperatures at which growth could still proceed slowly ranged between <30 °C and >34 °C [3,47,48]. The success of a species in a particular climate can depend on adaptation to either higher or lower average temperatures: duckweeds having a high optimum temperature (e.g., *S. polyrhiza*, *Le. aequinoctialis*, *Wo. globosa*) are better suited to warm climates, whereas those showing a lower optimum temperature (e.g., *La. punctata*, *Le. trisulca*, *Le. minuscula*, now *Le. minuta*) fare better in cooler and oceanic climates. Duckweeds exhibiting low minimum temperatures for growth (e.g., *Le. minor*, *Le. gibba*, *Le. trisulca*) have a better chance of survival in cooler climates, and those having a high maximum temperature for growth (e.g., *S. polyrhiza*, *Le. aequinoctialis*) will do well in more tropical surroundings [3]. The ability to tolerate very high temperatures over a relatively long period (e.g., 24 h at 50 °C and one week at 45 °C for *S. polyrhiza* [32]) is particularly advantageous for success in hot climates. Duckweeds must be able to react constructively to the heat stress that results when temperatures become dangerously high. Transcriptome analysis of the reaction of *S. polyrhiza* upon exposure to 45 °C demonstrated alterations in the expression of numerous genes, as well as increased superoxide dismutase activity parallel to malondialdehyde accumulation at the physiological level [49]. Exposure of *Le. minor* to 30 °C, which is a high temperature for this organism, resulted in DNA methylation that persisted over numerous frond generations and represents a long-term, transgenerational stress memory not observed in sexually reproducing plant species [29].

Very low temperatures that can threaten duckweed survival generally do not occur during the growing season. Some duckweeds can cope with the advent of such temperatures in the autumn by the formation of resting fronds, as is discussed in Section 5**.** The distribution of, e.g., *S. polyrhiza* in almost all climate zones [2,12] is a function of the high maximum temperatures for growth and the formation of turions upon the advent of cold winters exhibited by this species.

#### 4.1.2. Light

Duckweed growth and propagation are driven by the photosynthetic utilization of light, which is dependent on the temperature and nutrient and CO_2_ supply [47]. A duckweed requires sufficient light for suitable growth, whereby the highest rates of photosynthesis and growth possible for a particular species or clone take place at high light intensities. This is advantageous for the growth and propagation on open, unshaded water often observed for duckweeds. However, the maximum growth rates that can be achieved and the light intensities at which they are reached are strongly dependent on the temperature [47], and they vary considerably, depending more on the clone than on the species [48]. Duckweed success in growth and proliferation on a particular water body is thus not species-specific as much as requiring the presence of a clone well suited to the light intensity and temperature regimes at hand.

Very high light intensities can inhibit duckweed growth and damage the organisms, especially in terms of photoinhibition and oxidative damage [50]. The ability of duckweeds to grow rapidly at high light intensities depends on protective physiological features, such as the ability to convert much of the xanthophyll cycle pool to zeaxanthin and to dissipate much of the absorbed light non-photochemically, as shown by *Le. gibba* [51]. Growth can also be problematic at low light intensities, as in shading, in which case the light intensity required for light saturation and the compensation point of photosynthesis are important for growth. In a comparison of growth rates at different light intensities, clones of *Le. aequinoctialis*, *Le. valdiviana,* and *Le. minuscula* (now *Le. minuta*) showed the lowest optimum light intensity [47]. These clones would be expected to be the most shade tolerant, and the fact that they were collected from shady places illustrates that certain duckweeds can assert themselves well under limited light conditions. *Spirodela polyrhiza* responds to shading by increasing its frond surface area to optimize light capture, while *Le. minor* increases its chlorophyll content [52], and *Le. gibba* and *Le. minor* tolerate deep shade on the basis of large light-harvesting complexes and high photochemical efficiency [51]. The ability to grow better in shady conditions has the advantages of less exposure to high temperatures, better access to organic nutrient material (see following chapter), and usually quieter water conditions [2]. The advantage of a low compensation point for especially duckweed species that live below the water surface is illustrated by the occurrence of *Le. trisulca* at a depth of 12–14 m [53]. *Le. gibba* and *Le. minor* are exceptional in that their pronounced growth potential combined with pigment and photochemical characteristics of both shade and sun plants enables them to thrive under a wide range of high light intensities and ensures their success in dynamic light environments [51].

Duckweeds possess a differentiated cuticle to interface both the atmosphere on the adaxial side of the fronds and the water surface on the abaxial side. The biochemical composition of the cuticular waxes of *S. polyrhiza* is unique and may be of particular importance for the protection of the duckweed fronds under high light intensities, as it consists of up to 60% phytosterols that are important in the absorption of UV radiation and the scavenging of UV-generated radicals [54].

#### 4.1.3. pH Value

Many duckweeds are able to grow well at pH values of between 5 and 8 [3], although duckweeds have been found in natural waters with pH values between 3.5 and 10.4 [2]. Species found in nature at pH < 5 include *Le. minor*, *Le. aequinoctialis,* and *Wo. globosa*, and those observed at pH > 9 include *S. polyrhiza*, *Le. minuscula* (now *Le. minuta*), and *Wo. brasiliensis* [2]. Three species (*La. punctata*, *Le. minor,* and *Wo. arrhiza*) have been shown to tolerate pH values of up to 10 in laboratory experiments [55]. The lower pH value limits for the growth of essentially all duckweed species range between 3 and 4. A few species, such as *La. punctata*, *Le. turionifera,* and *Le. perpusilla,* can grow at pH 3.2–3.5, whereas others, including *S. polyrhiza*, *Le. trisulca,* and *Wa. hyalina,* cannot tolerate pH values of less than 4 [2]. The success of duckweeds in growing and proliferating on waters with especially extreme pH values can accordingly be dependent on the ability to tolerate these values. As an example, pH values above 8 have been reported to preclude both *Le. minor* and *Le. trisulca* growth [56] and thus cause local and temporal extinctions in the populations of these two species that are otherwise widely distributed in southern Ontario lake waters [31].

High temperatures, light intensities, and pH values can all disrupt duckweed growth and propagation and can, as such, be seen as stress factors that can induce flowering to ensure survival by the setting of viable seeds (see Section 6).

### 4.2. Ensuring Sufficient Nutrition

#### 4.2.1. Ensuring Mineral Salt Uptake and Storage

As facultative photoautotrophic organisms, duckweeds must have access to sufficient mineral ions, especially those of nitrogen, phosphorus, and sulphur. The mineral requirements of duckweeds have been summarized by Landolt and Kandeler [3]. Since phosphate (Pi) was the limiting mineral factor for floating aquatic plants under natural conditions in the pre-anthropogenic era, duckweeds, along with other macrophytes, have evolved to be particularly proficient in assimilating and storing this ion [9]. The priority of Pi uptake for duckweeds is illustrated by the uncoupling of this uptake from growth, i.e., the maintenance of Pi uptake by *Le. minor*/*japonica* at temperatures too low for growth [57].

Pi, which is the form of phosphorus usually taken up and assimilated by duckweeds [3], is made available to the plants by the action of phosphatases, which release Pi from organic material. Pi deficiency in the medium has long been known to inhibit the growth of and have other far-reaching effects on *S. oligorrhiza* (now *La. punctata*) [58], including strong enhancement of phosphatase activity [59]. Phosphatases and ribonucleases induced by Pi deficiency were observed in membrane-bound form at the water–plant interface and as exuded soluble enzymes [60,61]. The major phosphatase induced by low Pi supply in *S. oligorrhiza* (now *La. punctata*: [62]) was shown to be a glycosylphosphatidylinositol-anchored membrane protein [63] that was purified [64] and characterized as a purple acid phosphatase (PAP: [65]). The activity of this alkaline phosphatase may complement the induction of a high-affinity Pi transporter in the plasma membrane of *La. punctata* [66] in effecting the highly enhanced Pi uptake activity shown by this species under phosphate deficiency. The synthesis of PAPs and high-affinity Pi transporters are features of the PSR for optimizing external Pi acquisition.

*La. punctata* can store assimilated Pi in the vacuole as a reserve for growth upon the onset of Pi deficiency in the medium [67]. Linear oligophosphates and cyclic metaphosphates can function as short-term Pi reserves in *Le. minor* [68] and phytin as a long-term reserve in *Le. gibba* [69].

Plants can acclimatize to extended periods of Pi deprivation by eliciting a complex array of morphological, physiological, and biochemical/metabolic adaptations collectively known as the Pi-starvation response (PSR). The PSR arises in part from the coordinated induction of Pi-starvation-inducible genes encoding enzymes that reprioritize internal Pi use and maximize external Pi acquisition [70]; it may be stimulated in *S. polyrhiza* by SPX genes that are expressed in response to Pi (and nitrate) deficiency stress [71]. Interestingly, in this regard, starch accumulation—which is an expression of limited interior Pi availability—is strongly induced in duckweeds by deficiency of the mineral nutrient elements nitrogen and phosphorus [22], as well as sulphur [72], in the medium. Starch accumulation due to mineral nutrient deficiency is important in the formation of resting fronds and especially turions for overwintering (see Section 5). The accumulation, which may represent a depot of carbohydrate skeletons for use when mineral salts become more available again, reflects a reprioritization of available interior Pi. The accumulation of starch by Pi-deficient plant cells may largely arise from the release of ADP-glucose pyrophosphorylase, the gateway enzyme of starch synthesis, from allosteric inhibition by Pi, owing to the large reductions in cytoplasmic Pi pools that accompany long-term Pi deprivation [70]. Indeed, high starch accumulation in *La. punctata* has been shown to be a function of high-efficiency Pi recycling [73]. In addition, Pi and nitrogen deficiency were shown to increase the expression of starch-synthesizing enzymes [74] in addition to Pi transporters and phosphatases [73].

#### 4.2.2. Diet Supplementation with Organic Material

Although duckweeds generally grow photoautotrophically, using light and mineral salts for photosynthesis, they can also grow mixotrophically in light with sugars and even heterotrophically in the dark if sufficient sugars, amino acids, and vitamins are available in the medium [2,3,47,75]. The ability to transition between different trophic conditions was shown to endow *S. polyrhiza* with great metabolic flexibility [76]. Duckweed mixotrophy and heterotrophy are of commercial interest in the context of the production of starch-rich biomass [72], and especially mixotrophy is thought to be much more widespread in nature than previously thought [77]. Lake waters have been shown to contain sugars and other organic substances, and especially duckweeds living in shaded habitats such as *Le. trisulca* can supplement their photoautotrophic nutrition by the uptake of such substances [2]. Large amounts of organic substances can emanate from aging and dying water organisms, including duckweeds themselves when these form thick mats covering the water surface [9].

Mixotrophic nutrition requires the possession of the necessary systems for the uptake of organic substances, and the ability to compete effectively with ubiquitous aquatic microorganisms in assimilating organic substances from the medium. *Le. gibba* was shown to possess a constitutive active hexose uptake system [78], *Le. aequinoctialis* fronds have been shown to dispose of multiple carriers for taking up a large variety of small organic molecules against concentration gradients [79,80], and high-affinity transport systems for neutral/acidic and basic amino acids were described for *S. polyrhiza* [81]. Organic substances in the vicinity of the duckweeds are conserved by the release of phenolic substances. As shown for *La. punctata*, a number of flavonoid substances leach out into the medium from aging and dying fronds that exhibit antibacterial activity. Intact fronds also exude phenolic substances, as shown for *S. polyrhiza* and indicated for some other species [9].

### 4.3. Protection against Microbial and Insect Damage

Duckweeds have adapted to be able to thrive in aqueous environments rich in organic materials, as illustrated by their ability to grow on organic wastewaters (e.g., [23]) and their value in the remediation of such waters [10,11]. These environments can accordingly have a high microbial load, and since plants have bacterial virulence factors in common with animals, duckweeds are susceptible to microbial attack. This has been illustrated in the development of *Le. minor* as a model plant system for studying human microbial pathogenesis, with which *Staphylococcus aureus*, *Pseudomonas aeruginosa,* and several other bacteria known to be pathogenic to humans were shown to be severely detrimental to duckweed growth and viability [82]. However, the ability of duckweeds to tolerate highly microbial surroundings indicates that they may have particularly effective disease resistance function [4].

It is not clear how duckweed plants persist in a wide range of environments in the light of their susceptibility to bacterial phytopathogens in an experimental context. However, genetic analysis has shown that duckweed defence responses against pathogens differ from those of most plants [4]. *S. polyrhiza* and especially *Wo. australiana* contain significantly fewer of the nucleotide-binding leucine-rich repeat domain genes (NLRs) that encode many disease-resistant proteins than do other plant model organisms, which indicates that they do not require a large variety of NLRs for pathogen immunity and survival. Nevertheless, NLR genes are more important for the pathogen response of *S. polyrhiza* than of *Wo. australiana*, in which pattern-recognition receptors (PRRs) may play a more dominant role. Genes encoding the antimicrobial proteins (AMPs), lipid transfer proteins (LTP), defensins, and snakins were indicated to be vital for the pathogen resistance of the duckweeds. These findings were complemented by the determination that duckweeds lack the enhanced disease susceptibility gene ESD1 responsible for inducing anti-pathogen defence in most plants and that they feature the upregulation of AMPs absent from the model plant *Arabidopsis thaliana* upon pathogen attack [83].

Aquatic plants can be exposed to saprophytic and parasitic bacteria and fungi in the water that exude enzymes capable of degrading certain components of the plant cell walls. Duckweeds are protected against such microbial attack to an extent in that the composition of their cell wall substances differs considerably from that of most plants and is characterized by high contents of apiose and xylose [9]. Duckweeds (*Le. minor* and *Wo. arrhiza*) have long been known to be rich in apiose [84], which was found to be a component of the cell wall in *Le. gibba* and *Le. minor* [85]. The cell wall polysaccharide apiogalacturonan has been detected only in duckweeds and seagrasses [86,87]. In *Le. minor,* it has been found to contain about 25% apiose with some xylose [88,89], and the apio- and xylogalacturonans of the cell walls of *S. polyrhiza*, *Le. gibba,* and *Wo. australiana* constitute 48–57% of the cell wall mass of these species [90]. A substantial fraction of the apiogalacturonan fraction of *Le. minor* cell walls with a high apiose content was resistant to pectinase degradation, illustrating how apiose may protect pectic substances from the cell wall polysaccharide-degrading action of infecting pathogens [91]. Xylose and possibly also arabinose may have a function in the cell walls of duckweeds complementary to that of apiose. This is shown by the finding that the apiogalacturonan content in *Wolffiella* and *Wolffia* cell walls is far lower than that in *Spirodela*, *Landoltia,* and *Lemna* cell walls, but xyloglacturonan is far more abundant than apiogalacturonan in *Wolffia* cell walls, and *Wolffiella* cell walls have a high arabinose content [92].

Chemical defence strategies may also be involved in the response of duckweeds to pathogens. Cell extracts of *Le. minor* have been shown to have antibacterial and antifungal properties in that they inhibited the growth of strains of several bacterial and fungal species isolated from human patients, foods, or fish that can be pathogenic to humans or animals [93,94,95]. However, it is not clear if the extracted compounds that were detrimental to the microbes in biotests are actually involved in the resistance of intact duckweeds to pathogens. Flavonoids are well known to contribute to pathogen resistance in plants [96], and duckweeds contain large numbers of these compounds [97]. The effect of flavonoids on the duckweed weevil provides evidence that these substances can indeed be important in protecting duckweeds from biotic attack. *Le. minor* contains appreciable amounts of the flavones isoorientin, vitexin, and isovitexin that significantly decrease the survival rate of the larvae of the herbivore insect *Tanysphyrus lemnae* that feeds on the duckweed [98].

### 4.4. Cooperation with Microorganisms

Aquatic microorganisms do not only pose a threat to aquatic plants: they also engage in mutually advantageous cooperation with the macrophytes. Plants quite generally host structured communities of microorganisms, or microbiomes, that confer fitness advantages, including growth enhancement, nutrient uptake, stress tolerance, and pathogen resistance to the host [99]. Duckweeds have long been known to bear epiphytic bacteria on their fronds and roots [2], and more recent studies have revealed that their microbiome can stimulate growth, improve the removal of nutrients, heavy metals and xenobiotics from waters, and inhibit gas release from aquatic communities [91]. This has stimulated great interest in the duckweed microbiome in terms of optimizing duckweed biomass yields for the production of biofuel and improving duckweed-mediated water remediation. In conjunction with the advantages provided by its small size, rapid growth, ease of cultivation and analysis, and increasing genomic resources, duckweed has become a promising model organism for investigating plant–microbe interactions in aquatic environments [100,101,102].

A total of 24 genera of bacteria of the phylum Proteobacteria (now Pseudomonadota) constitute a highly consistent core microbiome over the four duckweed genera *Spirodela*, *Landoltia*, *Lemna,* and *Wolffia* [103]. An important point of inquiry is how such a microbial community is assembled. Microbiomes of *S. polyrhiza* and *Le. minor* collected at different locations were determined, and their similar compositional profiles—including the predominant Proteobacteria—were established even when surface-sterilized fronds were exposed to wastewaters quite different to the waters of their original habitats. In addition, these profiles were quite similar to those of the leaves of terrestrial plants [102]. This indicates that duckweeds actively assemble and maintain their microbiomes in a manner conserved among all plant leaves. Further investigation of microbiome assembly can be carried out with duckweed-based synthetic microorganism communities.

The association of bacteria with the duckweed frond is an important factor in the ability of the duckweed to survive or thrive in a given aqueous environment. If a duckweed associates with bacteria that increase its innate growth potential, it will have an enhanced ability to colonize open water and compete with other surface macrophytes for space, light, and mineral resources. The first plant growth-promoting bacterium (PGPB) identified was a strain closely resembling *Acinetobacter calcoaceticus* isolated from *Le. aoukikusa* (now *Le. aequinoctialis*) that was able to enhance the growth rate of the host duckweed while degrading phenol present in the medium [104]. Subsequently, numerous studies have been carried out for the improvement of duckweed yield by the application of PGPBs such as strains of *Sinorhizobium*, *Exiguobacterium* [100], *Pseudomonas* [105], and *Acidobacter* [106] in addition to *Acinetobacter*. Duckweed/bacteria associations can give rise to mutualistic growth promotion. The association of *Le. gibba* and an *Acinetobacter* strain resulted in the promotion of the growth of both the bacterium the duckweed [107]. This was also the case with the association of the nitrogen-fixing bacterium *Azotobacter vinelandii* and *Le. minor*. The bacterium provided growth promotion factors and fixed nitrogen for the duckweed, which enhanced the nitrogen-fixing activity and the cell number of the bacterium [108].

The probability of establishing a productive PGPB/duckweed association depends on the ability of the bacteria to attach to and remain adhered to the macrophyte. A strain of the PGPB *Aquitalea magnusonii* isolated from *Le. minor* proved to be very successful in colonizing the duckweed even in the presence of much higher titres of growth-inhibiting bacteria that also associate with the duckweed [109]. However, the growth-improving effect of the addition of a PGPB was—as has often been observed—only short-lived, due to the strong resilience of the natural duckweed microbial community [110]. If PGPBs play a role in duckweeds under natural conditions, they may be water constituents that temporarily attach and adhere to the duckweed or remained attached as components of the natural microbiome. Several bacteria in pond water attached to axenic *Le. minor* and were able to promote the growth of the duckweed [105].

The microbiome of duckweeds can help the macrophytes to improve the quality of their medium. The bacteria of the microbiome can assist in the removal of excess nutrients, heavy metals, and organic xenobiotics from the aqueous surrounding of the duckweed [100]. Recent examples are the synergistic action of *Le. gibba* and an *Acinetobacter* strain in removing ammonium nitrogen from aquaculture water [107], the identification of six bacterial strains adhered to *Le. minor* that could all efficiently remove phenol from the medium as well stimulate the growth of the duckweed [111], and the improvement of tolerance of *S. polyrhiza* to cadmium by the action of rhizobacteria native to the duckweed roots [112].

The microbiome can also respond constructively to changes in the environment. The relative abundance of many of the bacteria constituting the core microbiome of *Spirodela*, *Landoltia*, *Lemna,* and *Wolffia* species underwent marked changes upon the onset of nutrient deficiency in the medium, corresponding to indications of increased motility, biofilm formation, nitrogen metabolism, and biodegradative ability of the microbiome [94]. The presence of the PGPB *A. magnusonii* mitigated the inhibitory effect of copper and zinc on the growth of *Le. minor* and enhanced the duckweed’s ability to accumulate and tolerate these heavy metals [113]. Although this may not reflect processes occurring in nature, it illustrates how the duckweed microbiome interacts in a clonally dependent manner with environmental factors [114].

The microbiome can also influence the resistance of a duckweed to herbivory. Three of six different genotypes of *S. polyrhiza* inoculated with microbiota associated with the duckweed growing outdoors exhibited increased resistance by up to 41% to feeding by the pond snail *Lymnaea stagnalis*. However, three other genotypes showed *decreased* resistance to the herbivore attack [115], illustrating how clonal differences complicate the interpretation of duckweed cause/effect relationships, and that a beneficial effect on one clone may not be mirrored in another clone of the same species.

### 4.5. Coping with Water Pollution

Duckweeds grow on quiet or only slowly flowing waters, which are susceptible to contamination by numerous organic and inorganic substances from municipal, agricultural, and industrial wastewaters and run-off from fertilized fields. Many of the contaminating substances are toxic to duckweeds, and indeed duckweeds—especially *Le. minor* and *Le. gibba*—have long been used as test organisms in established test protocols for testing toxicity to aquatic higher plants [10,116]. The effects of water contaminants on duckweeds are illustrated by the biomarkers of effect that result from exposure to these substances [117,118]. These biomarkers can, on the one hand, show the harmful effects of water contaminants on a duckweed and can also, on the other hand, illustrate how the duckweed reacts constructively to the harmful influence of the contaminant to improve its survival chances in the presence of the contaminant.

Water contaminants can be classified into three groups: excess nutrients, metals, and organic xenobiotics. Nutrient water contaminants encompass the plant macronutrient ions NH_4_^+^, NO_3_^−^ PO_4_^3−^, and SO_4_^2−^ that can accumulate in surface waters from fertilizer washout and microbial action on organic wastewater. Contaminating metals comprise mainly heavy metals in dissolved ionic form or suspended as nanoparticles, as well as the metalloids As and Se, from industrial wastewaters and, to some extent, geological and solid waste leaching. A multitude of organic xenobiotic substances, including industrial chemicals, natural toxins, pesticides, pharmaceuticals, and personal care products, can also contaminate water. These myriad water pollutants detrimentally affect duckweeds on developmental, morphological, anatomical, physiological, biochemical, and molecular levels. Duckweeds respond to excessive nutrient supply with exaggerated growth leading to eutrophication, whereas other contaminants usually result in growth inhibition. Exposure to some metals can lead to frond disintegration, chloroplast damage, and frond starch accumulation. Oxidative damage due to the production of reactive oxygen species is very widespread, especially in conjunction with inhibition of photosynthetic activity and damage to the photosynthetic apparatus. These and numerous further observations of biochemical and molecular effects due to water contaminants are documented in Ziegler et al. [118]. In addition to the determination of specific biomarkers of effect, comprehensive transcriptomic analyses have illustrated the far-reaching differential gene expression and metabolic alterations occasioned by the deleterious effects of NH_4_^+^ [119], Cd^2+^ [120], and streptomycin [121] on *Le. minor*, *La. punctata,* and *Le. aequinoctialis*, respectively.

Duckweeds can react to alleviate damages caused by water contaminants. Several responses to deleterious impingement of water pollutants include increased activities of antioxidant and detoxification enzymes and enhancement of thiol protectant, flavonoid, phytochelatin, and heat shock protein synthesis (numerous examples in [118]). Such responses and the widespread physiological and molecular reactions to water contaminants revealed in the transcriptome studies mentioned above cannot be regarded as being duckweed-specific but are rather representative of remediative measures common to higher plants deleteriously affected by toxic substances. The formation of pectinous cell wall thickenings in *Le. trisulca* mesophyll cells that sequester lead taken up by the duckweed is an example of a widespread strategy in many plants to compartmentalize accumulated heavy metals away from sensitive sites in the protoplast [122]. Nevertheless, they illustrate that duckweeds can cope with water pollution as well as other plants to the extent that it does not prove to be too debilitating. However, a physiological and transcriptomic analysis of salt stress in *S. polyrhiza* revealed some mechanisms with respect to particularly hormone-related responses to salinity that appear to be different from those operative in other plants [123]. This may signify that duckweeds do have some unique means of coping with water pollution.

The coexistence of different duckweed species can be of mutual advantage to the involved organisms in coping with heavy metal stress. Both *S. polyrhiza* and *Le. aequinoctialis*, which frequently occur together in nature, grown together grew more rapidly when exposed to various concentrations of a mixture of copper, cadmium, and zinc than when grown separately. This was accompanied by an increase in the activities of antioxidant enzyme activities in both species, which increases tolerance to the metals. Metal uptake was thereby not limited so much as differentially accumulated: *Le. aequinoctialis* accumulated Cd and Zn preferentially, whereas *S. polyrhiza* accumulated mainly Cu and Cd [124]. In another study with the same two duckweed species, the presence of *S. polyrhiza* improved the growth of *Le. aequinoctialis* at high copper concentrations and decreased the environmental load of the heavy metal by increasing sequestration of Cu in the cell walls of *Le. aequinoctialis* [125].

The ability of duckweeds to withstand the deleterious effects of metals can be improved by the presence of growth-promoting bacteria that associate with the duckweed (see Section 4.4). An example is the alleviation of the harmful effect of chromium (Cr(VI)) on *Le. minor* in the presence of the rhizobacterium *Exiguobacterium* sp. MH3 by enhancing the growth of the duckweed and preventing the duckweed from taking up excessive amounts of the metal [126]. The presence of the PGPB *A. magnusonii* mitigated the inhibitory effect of copper and zinc on the growth of *Le. minor* and enhanced the duckweed’s ability to accumulate and tolerate these heavy metals [39]. The alleviation of the multiple heavy metal toxicity by the coexistence of *S. polyrhiza* and *Le. aequinoctialis* described above was accompanied by increased duckweed-associated microbial activity compared with that exhibited by the duckweed by itself and is indicative of regulation of the activities of the bacterial communities associated with the individual species [127].

Duckweeds may protect themselves from the harmful effects of water contaminants in water by degrading the toxic substances to non-toxic forms with the aid of bacteria in their microbiome. This has been illustrated by the colonization of sterilized *Le. aoukikusa* (now. *Le. aequinoctialis*) roots by a phenol-degrading *Acinetobacter* strain P23 that was isolated from the rhizosphere of the duckweed. A long-term continuous degradation of phenol in the medium was attributed to the beneficial symbiotic interaction between the duckweed and the bacterium [104].

Duckweed communities may experience pulse—in contrast to long-term—exposure to harmful water contaminants, following which surviving members of the community may recover to regain their original vitality and distribution. Both *Le. minor* and *Le. gibba* suffered significant inhibition of growth rate and biomass production upon exposure to >100 mg/L diuron for 7 days, after which they recovered completely when transferred to non-contaminated medium. This suggested that duckweed can withstand short-term exposure to environmentally relevant concentrations of herbicides at significant risk levels [128].

There is evidence that duckweeds may actually be able to develop resistance to herbicides such as diquat, which is used to control *Le. minor* and *Wo. columbiana* spreading in an unwanted manner [129]. *La. punctata* was found to be very susceptible to diquat if it had not previously been exposed to the herbicide but quite resistant if it had a prior history of exposure to diquat [130]. This also illustrates the ability of a duckweed to overcome anthropogenic management efforts to suppress it and thus increase its chances of survival.

A truly duckweed-specific means of coping with the presence of a heavy metal water contaminant is the production of turions by *S. polyrhiza* upon exposure to cadmium at a concentration inhibiting the growth of the fronds ([131]; see also Section 5.2.1). In this way, fronds threatened by Cd^2+^ produced robust, dormant derivatives that can avoid the deleterious effects of the metal. It would be interesting to determine if this is a Cd-specific effect or if it reflects a general defensive response to exposure to heavy metals.

### 4.6. Competition

Duckweeds often occur together with other floating water plants (see [1,2]). If they are then to sustain themselves, they must be able to assert themselves in the face of competition from these other macrophytes, as well as from algae and cyanobacteria, for space, light, and nutrients. Their most basic “trump card” in this respect is their ability to grow and propagate themselves rapidly. This enables them to quickly cover any open-water space available to them and consolidate their areas of dispersion by forming multi-layered mats. Their rapid, surface-covering growth can deprive other photosynthetic aquatic organisms of space, light, and nutrients, thus diminishing their competitive ability (see [132]). This is illustrated by the designation of *La. punctata*, *Le. minor,* and *Wo. columbiana* as problematic weeds that overgrow waterways [129,130] and the prevention of weed growth in rice fields by the introduction of *S. polyrhiza* and *La. punctata* [133].

Excessive rapid growth can, however, also lead to intraspecific competition in duckweeds and a decline in vitality. When a duckweed proliferates rapidly for a long time in a confined area, the fronds will bunch together to form mats of various thicknesses after having initially covered the water surface. This overcrowding leads to growth inhibition and the production of smaller and more uniform but morphologically modified fronds in *Le. minor* [39] and *S. polyrhiza* [134]. Contact between previously separated fronds has also been observed to result in a burst of ethylene release in *S. polyrhiza*, *Le. gibba,* and *Le. aequinoctialis* [135]. The ethylene formation may cause crowding-associated growth retardation, as well as the promotion of aerenchym formation in *S. polyrhiza* and especially in *Le. gibba* providing the fronds with greater buoyancy to help them surface in crowded surroundings [9]. When overcrowding persists and growth stagnates, flowering/seed set and turion formation can provide possibilities for escape and renewed growth at more opportune times. Crowding has been found to enhance turion formation in *S. polyrhiza* (see Section 5.2) when this has been initiated [136]. It also inhibits the turion germination when it is still in effect when the turions have lost their dormancy [137], thus precluding a precocious return to the growth mode.

In some cases, the success of a duckweed in the face of a potential competitor is dependent upon the extent to which the environmental conditions are conducive to the growth of each species. Free-floating *Le. gibba* and the submerged, rootless hornwort *Ceratophyllum demersum* are both common in temperate eutrophic waters but are mutually exclusive. Sufficient mineral nutrient availability and a neutral water pH value favoured the success of the duckweed over the hornwort, whereas a low inorganic nitrogen supply and a high water pH value led to takeover by *C. demersum* [138]. The relative success of competing duckweed and non-duckweed species is not merely a matter of growth, however. In monitoring the presence, abundance, and growth rates of *Le. minor*, *Le. minuta,* and the water fern *Azolla filiculoides*, it was concluded that the distribution of the macrophytes did not associate with nutrient or light levels. Although *A. filiculoides* had the highest growth rate, it occurred least frequently, in contrast to *Le. minor*, which grew the most slowly but had the widest distribution. The ability to persist under winter conditions and to disperse after disturbances appeared to be the major determinant of competitive success [139].

Specific morphological and physiological characteristics can enable certain duckweed species to survive in regions not supportive of other Lemnaceae. An example is the ability of frost-sensitive *S. polyrhiza* fronds to survive freezing winter temperatures by developing frond derivatives (turions: see Section 5.2) that can withstand the cold season in comparison with equally frost-sensitive fronds of the otherwise very similar *S. intermedia,* which do not develop turions [38].

An important factor in the competition between duckweeds and other aquatic plants that is not based on growth success is allelopathy, or the ability of an organism to influence other organisms sharing the same habitat by means of exuding chemical substances. This has particular significance when the duckweed and its competitor have a similar ability to grow rapidly and have similar requirements for light and nutrients. In some cases, duckweeds appear to have a competitive disadvantage in cohabitation with non-duckweeds due to allelopathy. The ability of the water soldier *Stratiotes aloides* to compete successfully with *S. polyrhiza* was concluded to result from an allelopathic influence of *S. aloides*, resulting in an inhibition of frond production and concomitant induction of turion formation (see Section 5.2) in the duckweed [140]. The ability of the green alga *Cladophora glomerata* to dominate *Le. minor* was concluded to be due to the production of phenolic compounds acting in an allelopathic manner [132]. Nevertheless, the cessation of growth under the production of turions represents a means of coping with a competitive disadvantage, and *Le. minor* was also observed to form potentially allelopathic phenols in competition with *C. glomerata*. Indeed, another report has also indicated that duckweeds may have allelopathic potential in that extracts of *Le. minor* fronds show inhibitory activity on the root and shoot growth of several terrestrial plant species [141]. These authors also identified (3R)-(-)-hydroxy-β-ionone as the active ingredient of a *Le. minor* extract that inhibited the growth of cress [142]. However, these findings are no proof of the actual allelopathic activity of duckweeds.

Cyanobacteria compete with aquatic plants not only in terms of the removal of nutrients from the water due to their capacity for rapid growth but also because of the toxic substances, especially microcystins, that they excrete [143,144]. *Microcystis aeruginosa* is a widely distributed cyanobacterium that can have harmful allelopathic effects on duckweeds. Microcystins have been observed to inhibit the growth of *Le. minor* [145,146,147], *Le. gibba* [148], *La. punctata* [149], and *Wo. arrhiza* [146]. However, microcystin has not always been observed to detrimentally affect *Le. gibba* [150], and susceptibility to microcystin toxicity has been shown to be clone-specific in *Le. minor* [151].

Besides developing microcystin-resistant clones, duckweeds have some means of counteracting the competitive disadvantage resulting from microcystin action. As illustrated with *Le. gibba*, duckweeds can take up and detoxify the cyanobacterial toxin [143]. Although *Le. japonica* growth was inhibited by co-cultivation with *M. aeruginosa*, the presence of the duckweed also inhibited the growth of cyanobacterium, presumably by excreting allelopathic chemicals of its own [152]. The possibility of allelopathic duckweed competition against cyanobacteria is lent plausibility by the detrimental effect extracts of *La. punctata* on *M. aeruginosa* [153].

In contrast to cases of dominance or exclusion, two (or more) species may stably coexist with one another even though they have similar requirements for growth and would be expected to compete openly for dominance. An analysis of the widespread common presence of *S. polyrhiza* and *Le. minor* indicates that while this coexistence requires fluctuating environmental conditions, it is not primarily dependent on interspecific differences in such characters as thermal reaction norms or dormancy behaviour. Rather, it requires subtle niche differences causing negative frequency-dependent growth that acts consistently across environmental gradients [154].

## 5. Coping with Winter Cold: The Formation of Resting Fronds

Temperatures ranging from below 8 °C to about 17 °C are sufficiently low to completely prevent frond growth of various groups of duckweeds that inhabit regions exhibiting temperate to very warm growing seasons, and although most fronds can tolerate temperatures down to the freezing point or somewhat lower for at least short periods, they usually cannot withstand prolonged or severe frost [2,3]. Fronds of *Le. minor* and *S. polyrhiza* have been observed to survive even when encased in ice for a prolonged period [3,155], but duckweeds usually respond to the onset of winter cold by forming resting fronds.

Resting fronds are generally smaller and more robust than the fronds characteristic of the growing season and have fewer air spaces, as well as higher starch contents [2,3,4,9]. Their extremely reduced or completely arrested growth and propagation is key to the survival of duckweeds under extended periods of winter cold. The very low metabolic activity of the resting state enables the quiescent fronds to endure long periods of conditions inimical to growth and propagation. The formation of resting fronds and their subsequent “reactivation”, i.e., germination and sprouting to give rise to new, growing fronds when conditions improve at some later point, constitute a scheme of survival in a purely vegetative mode. The survival that these fronds convey under cold conditions is based on avoidance of severe freezing temperatures and tolerance of temperatures not significantly below the freezing point. Two principal types of resting fronds can develop.

### 5.1. Resting Fronds Still Capable of Growth

Some resting fronds basically resemble the “normal” fronds of the growing season, although they are generally thicker and fleshier in appearance than the latter. Despite their restricted metabolism, they can still grow and even reproduce slowly when the adverse conditions are not too severe [1]. They can resume normal growth and propagation when conditions improve.

*La. punctata*, *Le. perpusilla*, *Le. gibba*, *Le. minor*, most strains of *Le. aequinoctialis,* and some strains of *Le. japonica* form resting fronds capable of growth that remain on the water surface. This surface location generally renders them suitable for survival only in winters not characterized by freezing temperatures. They may indeed avoid the effects of such temperatures when these do occur, however, by being pressed beneath ice forming on the water surface or by remaining attached via stipes to the pouches of mother fronds that have died and sunk to the bottom of the water body [2].

*Le. trisulca*, *Wa. gladiata,* and *Wo. arrhiza* form resting fronds capable of growth that sink to the bottom of the water body on account of their density due to reduced air spaces and high starch content. In their submerged surroundings, they avoid severe frost temperatures that may be in effect at the water surface since the water temperatures on the bottom hardly go below the freezing point [2]. They thus provide for survival even in very cold winters.

It has recently been described that 90% of *Le. minor* fronds—which are generally thought to overwinter on the water surface—growing on a pond in Quebec, Canada, survived very cold winters beneath massive ice layers [156]. Since neither the anatomy nor the actual location of the fronds beneath the ice were investigated, it is unclear to which category of resting fronds this remarkable rate of survival can be attributed.

Little is known of the mechanisms involved in the formation of the resting fronds still capable of growth or about their resumption of “normal” growth when conditions improve. The developmental cycle of resting fronds has been thoroughly investigated only on the example of the turions of *S. polyrhiza*. Since the resting fronds still capable of growth resemble turions functionally [3], the principles elucidated with regard to *S. polyrhiza* turions may also be relevant for the formation and activation of these fronds.

### 5.2. Turions

Duckweed turions are resting fronds that emerge from meristematic pockets in the “normal” mother fronds giving rise to them. They separate from the mother fronds and sink to the bottom of the body of water on which the “normal” fronds grow on account of their density. They are particular examples of detachable, truly dormant modified green shoots that are widespread in aquatic plants [2,157]. According to Landolt [2], they are found in *S. polyrhiza*, *Le. turionifera*, some clones of *Le. aequinoctialis,* and many species of *Wolffia* (*Wo. brasiliensis*, *Wo. borealis*, *Wo. angusta*, *Wo. australiana*, *Wo. arrhiza*, *Wo. columbiana*, *Wo. globosa*). They also occur in *Wo. microscopica* (our unpublished observation). Duckweed turions are morphologically different from the “normal” fronds that give rise to them. The turions of *S. polyrhiza* and *Le. turionifera* are flat and rounded, while those of *Wolffia* are very small and spherical [2]. As is typical for turion-bearing duckweeds, the turions of *S. polyrhiza* have smaller air spaces, smaller vacuoles, thicker cell walls, and much more starch than the “normal” fronds giving rise to them [158,159,160].

**Figure 1:**
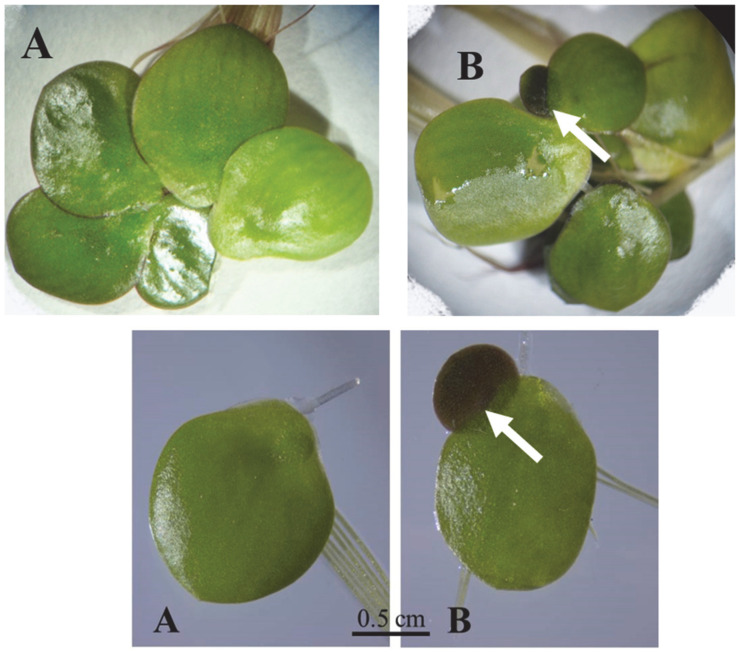
Fronds *of Spirodela polyrhiza* growing under non-limiting conditions (left-hand photos (**A**)) and under nutrient stress (right-hand photos (**B**)). The upper photos show colonies made up of several interconnected fronds, and the lower photos show single fronds that also exist alongside the multi-frond colonies. The fronds under nutrient stress produce dark turions are indicated by the white arrows.

Turions of *S. polyrhiza* (Figure 1) are more tolerant of low temperatures than are the “normal” growing season fronds of this species. However, this is not true of all duckweeds: turions of *Wo. arrhiza* are as sensitive to cold as are the “normal” fronds of this species [161]. Although turions cannot tolerate severe frost, they can withstand long periods of intense cold at the bottom of the water body where the water temperatures fall scarcely below the freezing point, as in the case of the submerged resting fronds still capable of growth. Turions are truly, or innately, dormant upon their formation in that they do not and cannot grow, although they do exhibit some respiration and are capable of photosynthesis [162]. Duckweed turions become capable of resuming growth once more after a prolonged period of exposure to low but not freezing temperatures. This “after-ripening” (turion formation can be termed “ripening”) breaks the dormancy and allows the turion to germinate and sprout to form new “normal” fronds when conditions again become conducive to growth and propagation [163].

The formation and overwintering of turions, as well as the subsequent germination and sprouting of these propagules to resume “normal” frond growth and vegetative propagation in the spring, has been thoroughly investigated only with *S. polyrhiza* (see [3]). The knowledge that has been amassed with this species is summarized below and provides a suitable picture of how turion formation enables a particular duckweed to survive cold winters.

#### 5.2.1. Turion Formation

Turion formation is a consequence of a switch in the developmental program of frond primordia from the formation of new fronds characteristic of the growing season to the production of resting turions [164]. In *S. polyrhiza*, a shortage of phosphate in the water is the prime environmental factor bringing about this switch, and low temperatures have the same effect when phosphate concentrations are higher [165,166]. The formation of turions is thus initiated in nature by the exhaustion of water resources at the end of a season of profuse aquatic plant growth and the approach of cold weather in the autumn. High light intensities and CO_2_ concentrations, as well as the presence of carbohydrates, can enhance turion formation in *S. polyrhiza* once this has been induced. This is due, however, to an increment in turion-producing biomass rather than representing a switch in the developmental program of the frond primordia [165,166] and is irrelevant for turion formation under natural conditions.

Turions are not formed exclusively in the context of overwintering. They can be produced upon phosphate deficiency at any time and may also be formed upon exposure to the heavy metal cadmium (see Section 4.5), as well as upon overcrowding and allelopathic influence (see Section 4.6). They can even be formed during the summer under conditions of high temperature and light intensity [167]. Turions can thus be seen as vegetative propagules formed in answer to various types of stress that must all act in a common or similar manner to re-program duckweed shoot primordia development. Abscisic acid is thought to be involved in this re-programming of *S. polyrhiza* turion formation [164,168,169].

The photomorphogenic effects of light (mediated by the photoreceptor phytochrome) can enhance or modulate turion formation in *S. polyrhiza* [170], but no critical day length, and thus no inductive effect of photoperiod, has been observed with this species [171]. It is remarkable that short days, which also herald the onset of the winter season, do not induce turion formation. Decreasing mineral nutrient availability in conjunction with decreasing temperatures thus gives rise to *S. polyrhiza* turion formation in nature in place of the low temperatures and short photoperiods usually responsible for turion formation upon the approach of winter in other hydrophytes [157,172,173,174].

Turion formation in *S. polyrhiza* shows great clonal variation when expressed as the specific turion yield (SY), i.e., the number of turions formed per frond under inductive conditions [25,175]. The SY is important in an ecological context as an indicator of the number of turions available to support the survival of the duckweed under adverse conditions, i.e., in winter [173]. Variability in SY represents adaptations to local climatic conditions and is presumably genetically determined [166]. The mean annual temperature of a site inhabited by an *S. polyrhiza* clone has an important influence on the SY of that clone. Low temperatures result in increased SY to offset the reduced survival rate of the turions under these conditions [25]. Clonal differences in turion formation as SY are independent of the specific signals that induce turion formation and are located in the transduction chain leading to the developmental switch from “normal” frond replication to turion production [173].

#### 5.2.2. Turion Dormancy

The innate dormancy that characterizes newly formed turions of *S. polyrhiza* is the key to the survival of duckweed in cold winters. Innately dormant turions in nature become able to germinate and resume normal vegetative growth after prolonged exposure to cold but not freezing temperatures (“chilling”). This “after-ripening” is a gradual response, the length of which depends on the conditions the turions are subjected to [3,32,176]. For *S. polyrhiza*, after-ripening must proceed for at least two weeks at water temperatures of 0–5 °C to remove the dormancy, as has been demonstrated by quantitative measuring the influence of the duration of after-ripening on the germination response [163]. This requirement for prolonged chilling ensures that the turion will not germinate or sprout precociously before the cold season has passed and conditions again become once more suitable for growth. *S. polyrhiza* turions may be formed in the late summer or early autumn in response to nutrient deficiency while temperatures are still warm and ample light is available. Without dormancy and the requirement of a protracted cold period to break it (i.e., resting on the bottom of the water body throughout the winter), the turions could germinate immediately after their formation with no prospect of appreciable growth and renewed turion formation before the onset of fatal winter water surface conditions.

The dormancy of newly formed turions represents a metabolic block, or state of “self-arrest” [177], that prevents the response of the turions to growth-promoting signals. It is not due to a lack of nutrient reserves to fuel metabolism, as the turions contain up to over 70% starch in terms of weight (e.g., [178]). However, this high carbohydrate reserve may initially not be accessible for turion metabolism. The prolonged dormancy of freshly formed turions may be related to a gradual breakdown of the highly polymeric starch molecules to soluble carbohydrates required for later germination metabolism. Freshly harvested *S. polyrhiza* turions indeed germinate to a certain extent, even without after-ripening in the presence of an external sugar supply [179]. Accordingly, newly formed turions may not normally contain levels of soluble, readily metabolizable carbohydrates sufficient to permit germination to take place. A gradual breakdown of the starch stored in newly formed turions has been observed to take place upon extensive storage of the turions under cold aqueous conditions [179]. Quantification of soluble sugars during turion after-ripening showed that this starch degradation resulted in the accumulation of soluble, readily metabolizable carbohydrates [180].

When after-ripened turions have lost their dormancy, they are in principle able to germinate in the presence of appropriate conditions of temperature and light. However, they will not germinate until these conditions actually apply. In their absence, the after-ripened turions remain quiescent in “imposed” dormancy (able to germinate but prevented from doing this by environmental constraints). This imposed dormancy persists after completion of after-ripening on the bottom of the water body until the water temperature has increased sufficiently to permit germination and ensure a successful resumption of growth.

#### 5.2.3. Turion Germination and Sprouting: The Resumption of Growth

##### Turion Rising: Bubble Formation

Turions that have waited out the cold of winter on the bottom of water bodies must surface in the spring to germinate and resume “normal” growth on the water surface to be able to re-establish themselves and propagate in their aquatic environment. How they do this is not clear, but submerged turions of *S. polyrhiza* have been observed to expel a small bubble of gas upon light incidence when the water temperature had increased to >15 °C. This bubble adheres to the junction between the pocket sheath and the upper surface of the turion and provides the turion with the buoyancy necessary to rise [32,181].

##### Germination

The actual resumption of growth commences with germination. “Germination” is the onset of developmental processes in quiescent turions as observed in terms of the reflection of leaves or scales and a slight elongation of the internodes [157]. The first indication of this in after-ripened *S. polyrhiza* turions is a slight swelling, after which 2 to 5 roots push through the root shield. When the first new shoot then pushes aside the pocket sheath as it emerges from the pocket, the turion is considered to have germinated. Germination normally begins shortly after the turions have reached the surface of the water and is dependent on temperatures of about 15 °C or higher and light [2].

Light has long been known to trigger turion germination [2,32], and the germination response of surfaced *S. polyrhiza* turions to light is mediated by phytochrome [182]. A single pulse of red light (“Rp”) induces germination: it can be reversed by a subsequent pulse of far-red light (“FRp” [182]) and is a low fluence-type, “classical” phytochrome response [183]. Germination can also be induced to a similar extent by repeated red light pulses or continuous red light (“cR”: [178,180]), which indicates a special low-fluence response that requires the presence of newly formed phytochrome in its far-red light absorbing, physiologically active form over an extended period [178].

Under natural conditions, germination is closely followed by sprouting, and the breakdown of the considerable reserves of starch stored in the turions (see [160]) would appear to be predestined to provide energy and carbon skeletons for the course of both developmental processes. However, germination can be induced by a red light pulse without starch breakdown and is, in this case, fuelled by soluble sugars having accumulated within the turion from the slow breakdown of storage starch during dormancy and after-ripening ([179,180]; see also Section 5.2.2).

##### Sprouting

Once turions have germinated, they “sprout” to resume vegetative growth, i.e., the production of new “normal” fronds. “Sprouting” commences with the distinct elongation of the still very short internodes of the germinated turions to enable better access to light, gas, and solute exchange for the emerging tissues, followed by the formation of new “normal” frond structures in the apical meristems (see [157]). Water temperatures favourable for germination (i.e., ≥15 °C) and light are key ecological requirements for turion sprouting.

Freshly germinated turions in *S. polyrhiza* are already equipped with effective photosynthetic and respiratory machinery [162], but the assimilative potential of the newly sprouted fronds is limited. Although a single red light pulse results in suitable germination of cold after-ripened *S. polyrhiza* turions, it leads to only very limited growth of the emergent sprouts. The weight of turions germinated in response to an Rp only doubled in the two weeks following the irradiation, whereas the growth of the newly emerging shoots progressed much more rapidly under cR irradiation [180]. This rapid sprouting is enabled by the breakdown of the reserve starch of the turions that is initiated by the cR treatment. The effect of cR in triggering *S. polyrhiza* turion starch breakdown lags only about 12 h behind germination, with the starch reserves of the turion being exhausted within a week [174]. Of course, sunlight in nature ensures both germination and starch degradation with its cR component.

The rapid mobilization of turion storage starch in nature occasioned by the cR component of sunlight thus provides young fronds emerging from turions upon germination with a supply of readily metabolizable carbohydrates sufficient to support the rapid frond growth and development of sprouting. This, together with the early surfacing and germination of after-ripened turions, is propitious for enabling the newly formed fronds to occupy the water surface before other plants in the spring.

#### 5.2.4. The Molecular Biology of the S. polyrhiza Turion Developmental Cycle

A very recent publication describes the results of an RNA-seq analysis carried out on mature turions and actively growing fronds from *S. polyrhiza* [184]. Differentially expressed transcripts between the mature turion and frond tissues revealed how the re-programming of frond meristems for turion formation involved the mobilization of major pathways related to the development of turion dormancy and to the starch and lipid metabolism that builds up nutrient reserves in the developing turions and remobilizes them again during turion germination and sprouting. It was also shown that dormant turions store numerous mRNA transcripts for use in mobilizing metabolic pathways required during the resumption of growth. DNA methylation appeared to represent an epigenetic component of turion tissue formation, and it was indicated that regulatory elements known to be involved in seed setting and germination have been reworked for analogous function in turions. This study provides a comprehensive conception of the molecular background of the turion-based overwintering strategy of *S. polyrhiza*.

#### 5.2.5. *Spirodela polyrhiza* as a Model for Turion-Based Duckweed Overwintering?

Experimental findings as to the developmental cycle of *S. polyrhiza* turions provide detailed insight into how one species of duckweed can survive winter cold by means of vegetative propagules that are formed under climatic conditions heralding the approach of winter, bridge long periods of low temperatures in a dormant state, and resume growth upon the onset of favourable conditions. The comprehensive picture might be regarded as a model for the overwintering of all duckweeds that form turions or functionally equivalent resting fronds. However, a model organism should be truly representative of a given set of organisms in a particular biological context, and very little information from other resting frond-bearing duckweeds is available for comparison with the extensive information pertaining to *S. polyrhiza*. Much further information along the lines of that presented here for *S. polyrhiza* must be gathered from these other species to evaluate how representative *S. polyrhiza* turions are for turion- or resting frond-based duckweed overwintering.

Of particular interest in this regard is how widespread the primary induction of turion formation by nutrient deficiency and low temperature evidenced in *S. polyrhiza* is. It is notable that Adamec [157] limited his comprehensive discussion of macrophyte turion physiology to mainly non-duckweed species on the grounds that turion formation in *S. polyrhiza*—as a representative of the duckweeds—was based on nutrient deficiency rather than the short photoperiods that are otherwise responsible for turion formation. If the formation of all duckweed turions is induced by mineral deficiency and low temperatures, this will represent a signature turion-based survival “strategy”.

## 6. Flowering and Seed Setting

Duckweeds do undergo sexual reproduction despite their more visible and widespread asexual vegetative propagation. Flowering and the production of viable seeds can always be a means for duckweeds to deal with situations inimical to growth and even life itself and is the only possibility of survival and reproduction when the duckweed habitat dries out completely or becomes too salty [9,185]. Flowering in duckweeds has long been of interest to researchers due to the fact that it is a question of the smallest flowering plants on Earth that are only rarely seen to flower when cultured under laboratory conditions. There have nevertheless been numerous observations of duckweeds flowering in the field, including all species except *Le. obscura*, *Wo. elongata,* and *Wo. australiana*. Some species flower relatively often (e.g., *Le. gibba*, *Le. perpusilla*, *Wa. lingulata*), whereas others do so only occasionally (e.g., *La. punctata*, *Le. minor*, *Wo. brasiliensis*) or very infrequently (e.g., *S. polyrhiza*, *Wo. borealis*) [2]. The actual frequency of flowering in nature may be higher than that observed, keeping in mind that a very small flower on a very small plant may be quite inconspicuous. Why particular duckweeds flower more frequently may not be easy to understand because many environmental factors can be involved in the induction of the flowering. These include crowding, light intensity and light duration, temperature, and the chemical composition of the water. Landolt [2] has tabulated the influence of these factors on the flowering of a number of duckweed species. In some locations, several duckweed species have been observed to flower at the same time, which indicates that environmental requirements may be similar for different species, but in other cases, flowering appears to be species-, season-, and location-specific [2].

Duckweeds have male and female floral organs, and two whorls of a typical flower—the sepals and petals—are missing. In the species belonging to the genera *Spirodela*, *Landoltia,* and *Lemna*, the floral organs develop into one of the two lateral pouches, normally giving rise to the vegetative buds that produce new fronds, whereupon the budding of daughter fronds from that pouch pauses. However, the daughter fronds continue to bud from the second lateral pouch present in these species. In the species belonging to the genera *Wolffiella* and *Wolffia*, the floral organs develop in a specialized cavity that opens to the dorsal side of the frond; the budding of daughter fronds thereby continues from the single vegetative pouch present in these species [2,18].

The sporadic occurrence of flowering and the ease of investigating duckweeds led to the use of duckweeds as a model organism for the investigation of flowering and have provoked numerous studies of flowering physiology and of the influence of environmental factors such as light, temperature, and the chemical makeup of the water in inducing the flowering [2,3,4,177]. Kandeler [186] rightly pointed out in 1984 that “Lemnaceae are one of the pilot systems to investigate the physiological basis of flowering”. The use of duckweeds in this respect features ease of maintenance and growth of gnotobiotic cultures in an aqueous medium, the uptake of investigatory chemicals directly from the aqueous culture medium, and the expeditious observation of effects on successive generations due to the rapid vegetative propagation of fronds [187].

**Figure 2:**
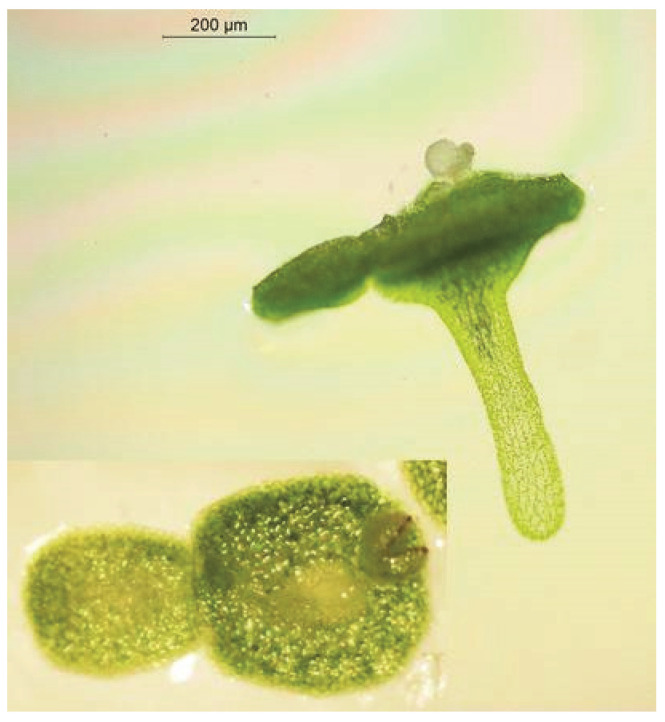
Flowering fronds of *Wolffia microscopica* with stigma and anther lobes seen on the exterior (lateral view). Inset: top view.

The geobotanical occurrence of the different species of duckweeds correlates with flowering behaviour as well as with growth. The differing photoperiodic and temperature requirements for the flowering of the various species of duckweeds are in coherence with the occurrence of the species in a widespread or a specific climate zone, e.g., day-neutral species exhibiting a cosmopolitan distribution or long-day species being distributed in the temperate regions [2]. Exposure to low temperatures (22 °C) induced flowering in *W. microscopica* [188,189] and has been able to induce flowering in this species even under continuous white light illumination (Sree and Appenroth, unpublished; Figure 2).

Several chemical compounds have been successfully used for initiating flowering in different duckweed species. The effects of molecules such as phytohormones and metabolites on flower initiation were investigated during the 1960s to 1980s [190,191]. Two compounds warrant special attention in this regard. Ethylenediamine-di-o-hydroxyphenylacetic acid (EDDHA) was shown to be a floral inducer in *S. polyrhiza* in 1966 [192], and 8 years later, the effect of salicylic acid (SA) on inducing flowering in duckweeds was established [193]. EDDHA and SA were thereafter successfully used for floral induction in several duckweed species under even non-inductive laboratory conditions [3]. EDDHA was able to induce flowering in plants sensitive to different photoperiods [192,194,195]. It was initially hypothesized that EDDHA acts by chelating metal ions that might be required for floral induction in duckweeds; however, it was subsequently suggested that the breakdown of EDDHA releases an SA-like active molecule that induces flowering [185,196]. With the current understanding of the role of SA in plant defence, Pieterse [185] suggested that flowering could be a stress response and that endogenously produced SA induces flowering upon exposure of the plant to stress conditions. Interestingly, crowding of plants, which is also a stress factor, has been suggested to induce flowering [2]. Crowding has been shown to increase ethylene production in *S. polyrhiza* [197], but Pieterse [198] found that ethylene did not induce flowering in *Le. gibba*. The bioassays originally planned for investigating florigen in duckweeds led to the identification of the floral-inducing capacity of SA [193,195]. Almost three decades later, the photoperiod-dependent flowering mechanism had been unfolded to a certain extent. The mobile florigen signal that is transported from the leaf to the shoot apical meristem has been identified as the FLOWERING LOCUS T protein (FT protein) that migrates through the phloem [199,200]. Two functional FT genes have been identified in *Le. aequinoctialis* that promote or suppress flowering [201], and the induction timing of an FT gene was shown to be important in connecting the phase of the circadian clock to photoperiodism at the molecular level in the same species [202]. These are important steps in understanding how flowering is initiated in duckweeds. The availability of ever more whole genome sequences of different clones and species of duckweeds has enabled the detection of the loss of five clades of MADS-box genes in duckweeds. This categorizes duckweeds as the angiosperms possessing the smallest number of clades of MIKC-type MADS-box genes [203]. Of the five, three of them, *AGL9*, *AGL12,* and *OsMADS32,* have been specifically lost in duckweeds. The authors have correlated this high number of missing clades to the simple architecture of the duckweed body and have suggested that the loss of *AGL9*-like genes may be responsible for the rarity of flowering in the world’s smallest angiosperms and, thus, for limited use duckweeds make of flowering and seed production to cope with untenable situations.

Flowering and the production of seeds is a strategy of duckweeds for the survival of drought or dry seasons. The seeds are able to tolerate desiccation on account of their anatomical structure, and they germinate upon the return of favourable conditions to develop into seedlings and establish a fresh duckweed culture in their environment [2]. It must be kept in mind, however, that induction of flowering does not ensure the production of viable seeds. Flowers may be aborted, or the floral organs may be sterile [204]. Pollination, which in nature can occur with assistance from wind, water, or small animals or by direct flower contact, must be successful, and self-pollination can result in sterility [1]. This is of present concern in the quest for the breeding of elite duckweed varieties for commercial applications and is being addressed by artificial cross-pollination [204].

## 7. Conclusions

Although they are very small, simply constructed, and apparently fragile aquatic higher plants lacking attachment to any substrate, duckweeds have proved to be very successful in colonizing new habitats and persisting on them in almost every part of the world. To do this, they cope with environmental conditions that are often less than optimal for growth and proliferation and may even prevent growth and be life-threatening. The means by which they do this reflects, in many instances, general patterns of plant response to environmental challenges. This is evident in how duckweeds adjust themselves to varied regimes of temperature, light, and pH value, ensure sufficient mineral and organic nutrient uptake, resist microbial and herbivore attacks, cooperate with microorganisms, and cope with water contaminants and competition for living space and nutrients during the growing season. The response of duckweeds to winter cold by forming resting fronds and turions is common to many macrophytes, and the flowering of duckweeds to survive life-threatening conditions is common to most higher plants. This indicates that duckweed responses to environmental stresses in the main reflect conserved survival strategies rather than unique mechanisms. Duckweeds do exhibit some highly specific survival characteristics, however, in terms of defence gene expression and cell wall composition in the face of microbial attack and the induction of turion formation by a mineral salt deficiency in place of photoperiodic effect otherwise evidenced by aquatic plants. However, the state of our knowledge about duckweed survival means is fragmentary: many relevant investigations have been carried out on only one or very few of the 36 duckweed species and have often not been carried out in depth. Conclusions about the extent to which responses of duckweeds as a plant family to environmental challenges have a unique status among plants are, therefore, premature.

What can be considered unique about the “survival strategies” of duckweeds is that much of the widespread success of these macrophytes can be recognized in an exceptional growth potential coupled with a primarily vegetative mode of frond propagation that gives rise to pronounced, epigenetically driven clonal diversity. The juvenile developmental status of the fronds that underlies the vegetative expansion also enables flowering and the development of overwintering propagules for surviving conditions that prevent growth and are potentially lethal.

In order to better understand the “survival strategies” of the family of duckweeds, future research must incorporate a more comprehensive selection of duckweeds into investigations of how these macrophytes react to environmental challenges. This may include comparing the findings respective of numerous duckweed species with those of selected “model” duckweed species to determine common duckweed traits and with those of established plant models such as *Arabidopsis* to assess how unique duckweed survival responses really are. This goal can be profitably approached by employing modern transcriptomic, proteomic, and metabolomic methods in the investigations wherever possible. In addition to increasing the understanding of duckweed responses to particular environmental stresses, the molecular information obtained with these techniques can identify via informatics how representative these responses are of general plant mechanisms.

## Data Availability

Not appliable.
